# Cross-sectional study on the impact of cardiac and hepatic iron overload, as measured by MRI T2*, on the quality of life in children with severe beta-thalassemia major

**DOI:** 10.1097/MD.0000000000038817

**Published:** 2024-07-05

**Authors:** Xiang Lan, Zhonglv Ye, Jiayi Du, Lili Liu, Chuan Tian, Linming Huang, Xiaohuan Mo

**Affiliations:** a Department of Paediatrics, Affiliated Hospital of Guangdong Medical University, Zhanjiang, China; b Department of Laboratory, Affiliated Hospital of Guangdong Medical University, Zhanjiang, China.

**Keywords:** beta-thalassemia major, cardiac iron overload, hepatic iron overload, MRI T2* imaging, quality of life

## Abstract

A cross-sectional study to explore the correlation between cardiac and hepatic iron overload and its impact on the quality of life in children diagnosed with severe beta-thalassemia major (β-TM). A cohort of 55 pediatric patients with β-TM, diagnosed via genetic testing at the Affiliated Hospital of Guangdong Medical University from January 2015 to January 2022, was included in this study. The assessment of cardiac and hepatic iron overload was conducted using the magnetic resonance imaging T2* technique. The Chinese version of the Pediatric Quality of Life Inventory (PedsQL) 4.0. Pearson correlation analysis was utilized to assess the relationships between the cardiac and hepatic T2* values and between these T2* values and the total scores of PedsQL 4.0. Analysis showed no significant correlation between cardiac and hepatic T2* values. However, a significant relationship was observed between cardiac T2* values and PedsQL 4.0 total scores (*r* = 0.313, *P* < .05), indicating that cardiac, but not hepatic, iron overload is associated with the quality of life. This study highlights the absence of correlation between cardiac and hepatic iron overload levels and demonstrates a significant impact of cardiac iron overload on the quality of life in children with β-TM. These findings suggest the need for a focused approach to cardiac health in managing β-TM.

## 1. Introduction

Iron overload represents a significant challenge in patients with beta-thalassemia major (β-TM), where the accumulation of excess iron necessitates implementing effective management strategies. Iron-chelation therapy plays a pivotal role in mitigating the detrimental effects on organ function, with a particular focus on cardiac health.^[1–4]^ Beyond its impact on cardiac and hepatic function, iron overload also adversely affects reproductive health and brain function, highlighting the critical need for a comprehensive evaluation of its widespread effects.^[5–7]^

The introduction of magnetic resonance imaging (MRI) T2* has transformed the noninvasive measurement of iron overload in diverse organs. The MRI T2* score serves as an essential diagnostic tool, reflecting iron deposition levels by gauging the relaxation time of protons within tissues. Decreased T2* times indicate elevated iron levels, rendering MRI T2* indispensable for evaluating and managing organ-specific iron loads in disorders such as β-TM. Its application extends across various medical conditions, including neurodegenerative diseases and diabetes, shedding light on the systemic implications of iron overload.^[8–10]^ Specifically, in β-TM, MRI T2* is instrumental in assessing iron content in cardiac and hepatic tissues, thereby facilitating targeted therapeutic strategies and forecasting clinical outcomes with greater reliability.^[11,12]^

Given the profound impact of iron overload on the quality of life among pediatric patients with β-TM, the Chinese version of the Pediatric Quality of Life Inventory 4.0 (PedsQL 4.0) has been validated as a crucial instrument for its evaluation. This tool is instrumental in investigating the association between cardiac and hepatic iron overload and quality of life, thereby identifying critical factors that negatively influence these patients’ well-being.^[13]^ In managing iron overload in β-TM patients, a holistic approach is essential, encompassing genetic factors, therapeutic interventions, and ongoing monitoring. In this regard, MRI T2* stands out as an indispensable resource, offering a comprehensive overview of iron distribution and its ramifications. This detailed insight aids in tailoring treatment approaches and enhancing patient outcomes effectively. This study aims to clarify the relationships between MRI T2* measurements, organ-specific iron burden, and quality of life in children with severe β-TM. It underscores the pivotal role of thorough iron overload evaluation in refining patient care strategies.

## 2. Methods

### 2.1. Participants

This study encompassed a cohort of 55 pediatric patients diagnosed with β-TM via genetic testing at the Affiliated Hospital of Guangdong Medical University between January 2015 and January 2022. The Ethics Committee of the Affiliated Hospital of Guangdong Medical University approved this research (Approval No. PJ2021-005). The cohort included 34 males and 21 females, aged between 6 and 14 years, with an average age of 10.7 years (standard deviation ± 2.37 years). Selection criteria focused on patients with a history of regular blood transfusions and iron-chelation therapy.

A comprehensive medical review was undertaken for each candidate to exclude any concurrent conditions such as heart failure, liver dysfunction, or malignancies, which could potentially skew the study’s outcomes. Additionally, inclusion required the patient’s ability to accurately complete the PedsQL 4.0 questionnaire, independently or with parental support. This ensured reliable and precise data collection via the Questionnaire Star platform, reinforcing the study’s methodological integrity.

### 2.2. Data collection and measures

This study’s exhaustive dataset was meticulously assembled for each pediatric patient diagnosed with β-TM. The dataset incorporated an array of variables: demographic information (age, gender), medical history (splenectomy status, frequency of blood transfusions), socioeconomic parameters (caregiver’s relationship and education level, family monthly income), personal and environmental considerations (number of siblings, personality traits, residential location), psychosocial factors (anxiety related to physical appearance changes, experiences of discrimination), and crucial clinical indicators (cardiac and hepatic T2* values). Moreover, the PedsQL 4.0 was utilized to quantitatively assess the patient’s quality of life, detailing the aggregate and individual domain scores.

A pivotal element of the research involved examining the association between cardiac and hepatic iron overload, as determined by T2* values, and the quality of life in these children. This integrated approach, encompassing a blend of clinical, psychological, and socioeconomic factors, was designed to unravel the comprehensive impact of β-TM on the lives of pediatric patients, aiming for an in-depth understanding of its extensive effects.

### 2.3. MRI protocol

Cardiac and hepatic T2* measurements in pediatric β-TM patients were conducted employing a GE Optima 360W 1.5T MRI scanner. Cardiac T2* imaging utilized electrocardiographic and respiratory gating to enhance accuracy. The protocol featured a single breath-hold 8-echo sequence, positioning the scan plane in the short axis between the apex and base of the left ventricle, ensuring a detailed assessment of myocardial iron load.

For hepatic T2* imaging, the IDEAL-IQ sequence was selected for its efficacy in covering the entire liver within a single breath-hold, thereby providing a comprehensive evaluation of hepatic iron levels.

The GE Advantage Workstation 4.6 facilitated the postprocessing and analysis of cardiac and hepatic T2* values. This methodical approach to imaging and analysis is essential for accurately quantifying iron overload in both organs, contributing significantly to an enhanced understanding of its implications for patient health and quality of life.

### 2.4. Outcome measures

To assess the quality of life in pediatric patients with β-TM, this study utilized a survey methodology employing the Chinese version of the PedsQL 4.0. Data collection was facilitated through an online deployment via the Questionnaire Star platform, ensuring convenient access for participants and their caregivers to complete the survey.

The PedsQL 4.0 instrument is an extensive measure that evaluates the quality of life across 4 critical domains: physical, emotional, social functioning, and school performance, comprising 23 items. Elevated scores denote improved quality of life, reflecting the instrument’s multidimensional assessment capability. This approach enables a comprehensive evaluation of the diverse facets of daily life impacted by β-TM, offering an integrated perspective on patient well-being that transcends mere physical health to include emotional well-being, social integration, and educational achievement. Adopting this multifaceted quality-of-life measurement is pivotal for understanding the extensive implications of β-TM on the lives of pediatric patients, aligning to delineate the condition’s expansive effects.

### 2.5. Diagnostic criteria for iron overload

In this investigation, we delineated specific thresholds for diagnosing iron overload in the heart and liver utilizing T2* MRI values: cardiac iron overload was determined when cardiac T2* values fell below 20 milliseconds (ms), and hepatic iron overload was recognized with hepatic T2* values <6.7 ms, following established references.^[14]^

Subsequently, patients were classified into 4 distinct groups for in-depth analysis: normal (absence of cardiac or hepatic iron overload), iron overload (presence of both cardiac and hepatic iron overload), cardiac (exclusive presence of cardiac iron overload), and hepatic (exclusive presence of hepatic iron overload). This classification facilitated a detailed examination of the varying impacts of iron overload on different organs and its association with quality of life, thus offering a thorough understanding of the clinical spectrum of iron burden in β-TM patients. Such stratification is essential for discerning the diverse effects of iron deposition across various organs and devising precise therapeutic interventions.

### 2.6. Statistical analysis

Statistical analyses in this investigation were performed utilizing SPSS Statistics version 25.0. Categorical variables were reported as percentages and analyzed using the Chi-square (χ^2^) test. Continuous variables were tested for normality; those adhering to a normal distribution were expressed as mean ± standard deviation. The independent samples *t* test facilitated comparisons between 2 groups for normally distributed data, whereas 1-way analysis of variance was applied for comparisons across multiple groups.

The median served as the measure of central tendency for data not conforming to a normal distribution. Pearson correlation analysis was applied to continuous data exhibiting a normal distribution, while Spearman rank correlation was employed for ordinal data deviating from normal distribution patterns.

The internal consistency of the PedsQL 4.0 was evaluated using Cronbach α coefficient. Additionally, confirmatory factor analysis was executed using AMOS version 25.0. This comprehensive statistical methodology underpinned a rigorous analysis, affirming the validity and reliability of the study’s conclusions regarding the impact of β-TM on quality of life and the magnitude of iron overload.

## 3. Results

### 3.1. Cardiac and hepatic MRI T2* results and correlation analysis

In this cohort of 55 pediatric patients with β-TM, cardiac MRI T2* values averaged 30.47 ± 15.04 ms, identifying myocardial iron overload in 16 patients (29%) with an average T2* of 11.77 ± 3.88 ms. This subgroup was categorized into 4 cases: mild, 7 moderate, and 5 severe myocardial iron overload. Hepatic MRI T2* values were recorded at an average of 2.75 ± 1.54 ms, with 52 patients (95%) demonstrating hepatic iron overload, with a mean value of 2.45 ± 0.90 ms. The severity distribution of hepatic iron overload was noted as 16 mild, 35 moderate, and 1 severe cases. No significant correlation emerged between cardiac and hepatic T2* values (*r* = 0.235, *P* > .05). As measured before MRI examinations, serum ferritin (SF) levels exhibited a mean value of 5156.66 ± 3410.99 ng/mL. A substantial proportion of the cohort, 52 patients (95%), had SF levels above 1000 ng/mL, with a mean of 5412.94 ± 3297.84 ng/mL. A notable negative correlation was found between serum SF levels and both cardiac T2* (*r* = −0.562, *P* < .05) and hepatic T2* values (*r* = −0.398, *P* < .05) (Table 1).

**Table 1:** Correlation of T2* values in cardiac, hepatic, and serum ferritin.

		Cardiac T2* values	Hepatic T2* values
Hepatic T2* values	*r*	0.235	
	*P*	.084	
SF	*r*	−0.562	−0.398
	*P*	<.001	.003

SF = serum ferritin.

This detailed analysis elucidates the extent and severity of iron overload in the cardiac and hepatic systems among pediatric β-TM patients. The absence of a significant interrelation between cardiac and hepatic iron levels highlights the imperative for targeted assessment of iron overload in these organs. Furthermore, the inverse relationship between SF levels and T2* values in both the heart and liver suggests the potential efficacy of SF as an indicative biomarker for iron overload in β-TM, warranting its consideration in clinical evaluations.

### 3.2. PedsQL 4.0 outcomes

The PedsQL 4.0 exhibited excellent internal consistency, with a Cronbach α of 0.89, underscoring its reliability. The subscales, encompassing physical, emotional, social, and school functioning, showed α coefficients ranging from 0.86 to 0.87, significantly surpassing the accepted reliability threshold of 0.7.

Critical metrics demonstrated strong structural validity in terms of construct validity: the χ^2^/df ratio stood at 1.58, the root mean square error of approximation was recorded at 0.08, and the fit indices: including the incremental fit index, Tucker–Lewis index, and comparative fit index: were observed at 0.91, 0.91, and 0.92, respectively.

The aggregate mean score on the PedsQL 4.0 reached 62.42 ± 15.99. Detailed subscale scores were as follows: physical functioning at 68.58 ± 15.79, emotional functioning at 62.64 ± 19.31, social functioning at 67.45 ± 18.68, and school performance at 51.00 ± 20.44. These scores elucidate the broad spectrum of quality of life challenges faced by children with β-TM, highlighting significant areas for intervention and support.

### 3.3. Correlation between MRI T2* values and quality of life

Cardiac T2* values exhibited a significant positive correlation with both the overall score (*r*  = 0.313, *P* < .05) and the physical functioning subscale (*r* = 0.445, *P* < .05) of the PedsQL 4.0. Conversely, hepatic T2* values did not significantly correlate with the PedsQL 4.0 total score or its subscale scores (*P* > .05). Similarly, SF levels were not significantly correlated with the PedsQL 4.0 total score (*P* > .05) (Table 2).

**Table 2:** Correlation analysis of quality of life and MRI T2* value.

	Cardiac T2* values		Hepatic T2* values	
	*r*	*P*	*r*	*P*
Total scores	0.313	.020	0.044	.749
Physical function	0.445	.001	0.108	.434
Emotional function	0.229	.092	0.003	.980
Social function	0.162	.239	0.011	.934
Role function	0.269	.051	0.041	.765

MRI = magnetic resonance imaging.

These findings underscore a meaningful association between cardiac iron accumulation and physical functioning in pediatric patients with β-TM, highlighting the detrimental impact of cardiac iron load on certain quality-of-life aspects. In contrast, hepatic iron load and SF levels exert minimal influence on the quality-of-life dimensions evaluated in this study.

### 3.4. Variations in quality of life across different groups

**Figure 1.**
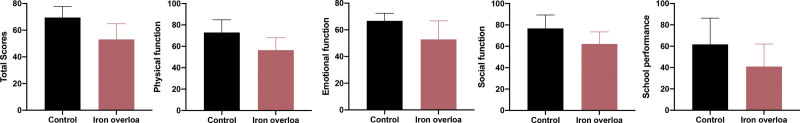
The difference analysis of quality of life between control and iron overload groups.

**Figure 2.**
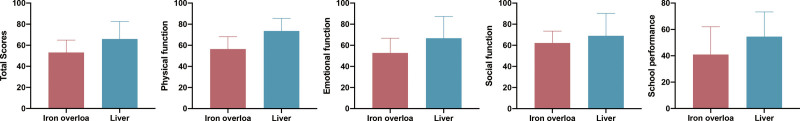
The difference analysis of quality of life between iron overload and liver groups.

The distribution of participants was categorized as follows: no iron overload group (3 cases, 5.5%), iron overload group (16 cases, 29%), cardiac group (0 cases), and hepatic group (36 cases, 65.5%). Examination of the PedsQL 4.0 total and domain scores revealed significant differences in overall scores among the groups (*P* < .05), with marked disparities observed mainly in the physical functioning domain (*P* < .05). Other domains exhibited no significant differences (*P* > .05) (Table 3). A comparative analysis between specific groups showed that both the no iron overload and iron overload groups and the hepatic and iron overload groups displayed significant variances in PedsQL 4.0 total scores (*P* < .05). Contrastingly, comparisons between the no iron overload and hepatic groups did not demonstrate significant differences (*P* > .05), suggesting cardiac iron overload more significantly impacts the quality of life. Significant differences were noted in physical functioning between the no iron overload and iron overload groups (*P* < .05) and physical, emotional, and role functioning between the hepatic and iron overload groups (*P* < .05). No significant differences were observed across all 4 domains between the no iron overload and hepatic groups (*P* > .05). The lack of participants in the cardiac group eliminated the possibility of inter-group comparisons (Figs. 1 and 2 and Table 4). This analysis delineates the complex influence of iron overload on various dimensions of quality of life, accentuating the differential effects of iron deposition in the heart and liver on the health and well-being of pediatric patients with β-TM.

**Table 3:** Difference analysis of quality of life in 4 groups.

	Total scores	Physical function	Emotional function	Social function	School performance
No iron overload groups	69.48 ± 8.30	72.92 ± 11.83	66.67 ± 5.77	76.67 ± 12.58	61.67 ± 24.66
Iron overload groups	53.10 ± 11.76	56.45 ± 11.66	52.81 ± 13.90	62.19 ± 11.25	40.94 ± 21.07
Cardiac group	0	0	0	0	0
Hepatic group	65.97 ± 16.56	73.61 ± 11.95	66.67 ± 20.70	69.03 ± 21.27	54.58 ± 18.72
*F*	4.393	8.521	3.152	1.134	3.128
*P*	.017	.001	.051	.330	.052

**Table 4:** Difference analysis of quality of life between no iron overload group and hepatic group.

	Total scores	Physical functioning	Emotional functioning	Social functioning	School performance
No iron overload groups	69.48 ± 8.30	72.92 ± 11.83	66.67 ± 5.77	76.67 ± 12.58	61.67 ± 24.66
Hepatic group	65.97 ± 16.56	73.61 ± 11.95	66.67 ± 20.70	69.03 ± 21.27	54.58 ± 18.72
*t*	0.360	−0.078	0.002	0.608	0.617
*P*	.721	.938	.999	.547	.541

### 3.5. Quality of life correlations with clinical and socioeconomic factors

Socioeconomic and caregiver-related factors significantly influence the quality of life in pediatric patients with β-TM. Noteworthy correlations include the educational level of caregivers, caregiver relationship, family per capita monthly income, concerns regarding appearance changes, and experiences of discrimination (*P* < .05). Conversely, demographic and clinical variables such as gender, age, personality traits, splenectomy status, transfusion frequency, and the number of siblings did not show a significant association with quality of life (*P* > .05) (Table 5).

**Table 5:** Clinical data distribution, difference, and correlation analysis of patients’ quality of life.

Factors	Numbers (%)	PedsQL 4.0 total scores	Quality of life		生存质量	
			*t*/*F*	*P*	*r*	*P*
Gender			0.002	.804	0.004	.739
Male	34 (61.8%)	62.84 ± 16.36				
Female	21 (38.2%)	61.73 ± 15.73				
Age			1.203	.278	0.168	.219
6–10	27 (49.1%)	64.82 ± 16.41				
11–14	28 (50.9%)	60.10 ± 15.50				
Splenectomy			0.140	.889	−0.044	.749
Yes	4 (7.3%)	61.33 ± 20.43				
No	51 (92.7%)	62.50 ± 15.84				
Transfusion frequency			0.675	.514	0.090	.512
≤2	13 (23.6%)	61.78 ± 14.95				
2–4	24 (43.7%)	60.14 ± 16.76				
≥4	18 (32.7%)	65.91 ± 15.92				
Caregiver			4.637	.014	0.379	.004
Father	8 (14.5%)	51.71 ± 15.98				
Mother	43 (78.2%)	62.82 ± 15.34				
Other	4 (7.3%)	79.49 ± 3.643				
Caregiver education level			3.335	.043	0.353	.008
Primary school	15 (27.2%)	53.79 ± 13.76				
Secondary school	37 (67.3%)	65.38 ± 16.29				
College	3 (5.5%)	68.90 ± 3.52				
Average monthly income			7.194	.002	0.491	<.001
<1000	16 (29.1%)	51.55 ± 15.87				
1000–3000	31 (56.4%)	65.40 ± 14.21				
>3000	8 (14.5%)	72.57 ± 11.79				
Sibling			0.967	.387	0.169	.218
0	5 (9.1%)	54.19 ± 8.15				
1–3	37 (67.3%)	62.31 ± 16.47				
＞3	13 (23.6%)	65.87 ± 16.49				
Character			0.063	.939	−0.007	.960
Introvert	11 (20.0%)	61.24 ± 20.23				
Extrovert	19 (34.5%)	63.36 ± 12.52				
Fall in between	25 (45.5%)	62.21 ± 16.89				
Residential address			0.419	.660	0.089	.520
Countryside	27 (48.1%)	60.58 ± 15.99				
Town	13 (23.6%)	65.48 ± 20.18				
City	15 (27.3%)	63.06 ± 12.07				
Appearance anxiety			3.483	.001	−0.429	.001
Yes	38 (69.1%)	57.84 ± 15.15				
No	17 (30.9%)	72.64 ± 13.07				
Discrimination or unfair treatment			3.483	.001	−0.481	<.001
Yes	17 (30.9%)	57.84 ± 15.15				
No	38 (69.1%)	72.64 ± 13.07				

### 3.6. Correlation analysis of cardiac T2* values with clinical data

Subsequent analyses delved into the relationship between cardiac T2* values and clinically significant factors identified earlier. These investigations revealed that cardiac T2* values do not significantly correlate with these socioeconomic and caregiver-related factors (*P* > .05). This outcome posits cardiac T2* values as independent predictors of quality of life in pediatric patients with β-TM, highlighting the cardinal role of cardiac iron burden.

This segment of the analysis accentuates the unique influence of cardiac iron load on quality of life, distinct from socioeconomic and caregiver-related factors. The lack of significant correlations further delineates the importance of cardiac T2* values in the nuanced clinical evaluation and management of β-TM, emphasizing their singular impact on patient well-being.

## 4. Discussion

β-TM, a chronic hemolytic condition, relies on standard transfusion and iron chelation as cornerstone treatments. These interventions, however, often culminate in iron overload, significantly affecting vital organs such as the liver and heart. The heart’s particular vulnerability to iron overload highlights a critical area of concern, as heart failure and severe arrhythmias emerge as leading causes of mortality in these patients.^[15,16]^ This situation underscores the imperative for meticulous monitoring of systemic iron accumulation.

MRI quantification has become an indispensable, noninvasive diagnostic technique for evaluating organ-specific iron overload. In our study, a notable prevalence of hepatic iron overload was observed in 98% of the children, predominantly presenting with mild to moderate severity. In contrast, cardiac iron overload was identified in 29% of the participants, indicating a temporal delay in cardiac iron deposition compared to hepatic deposition.

It’s crucial to investigate the relationship between MRI T2* values and the quality of life in pediatric patients with severe β-TM. Our analysis revealed no significant correlation between cardiac and hepatic T2* values (*r* = 0.235, *P* > .05).^[17]^ Challenging the utility of hepatic iron overload as a predictor for cardiac iron burden. While certain studies have suggested a correlation,^[18,19]^ discrepancies may arise due to variations in transfusion frequency and the effectiveness of iron-chelation therapy.

Moreover, SF levels are inversely correlated with cardiac and hepatic T2* values, echoing findings from several studies.^[20]^ Nonetheless, the link between SF levels and the risk of iron overload-related cardiac complications remains inconclusive, suggesting that while SF levels may reflect the overall iron burden, they may not reliably predict cardiac iron overload.^[21]^

Applying the Chinese version of the PedsQL 4.0 facilitated a detailed assessment of the quality of life among β-TM patients. The instrument’s utility is reinforced by its consistency with psychometric analyses from previous studies, providing a solid framework for evaluation.^[22–24]^ Notably, a positive correlation between cardiac T2* values and quality of life, particularly in physical functioning, was identified, highlighting the adverse effects of cardiac iron accumulation.^[25]^ Conversely, hepatic iron overload and SF levels did not significantly impact quality of life.^[26]^ The research further underscores the influence of socioeconomic and caregiver-related factors, rather than demographic or clinical variables, on quality of life.^[27]^ The widespread hepatic iron overload and the associated decline in quality of life emphasize the critical need to address socioeconomic obstacles to enhance patient outcomes.^[28]^

This exploration of MRI T2* values and their impact on the quality of life in pediatric patients with severe β-TM yields vital insights into the condition’s comprehensive effects. The findings highlight the necessity for holistic therapeutic strategies to navigate the intricate challenges faced by these patients, offering valuable perspectives on the broader implications of β-TM on patient health and well-being.

## 5. Conclusion

In conclusion, this study elucidates a pronounced discrepancy between cardiac and hepatic iron overload incidence rates in patients with β-TM. Notably, it underscores the lack of correlation between cardiac and hepatic T2* values, indicating that hepatic iron overload cannot be a reliable predictor for cardiac iron accumulation. Moreover, the investigation highlights that cardiac iron overload, a critical determinant of quality of life, is influenced by the frequency of transfusions. Deviations from standardized transfusion and chelation protocols may lead to the premature emergence of cardiac iron overload, markedly impairing quality of life. Consequently, strict adherence to established transfusion and chelation guidelines is paramount in clinical practice. For those patients demonstrating signs of cardiac iron overload, immediate adjustments to chelation therapy are vital. Such interventions are essential to mitigate the impact of iron overload on the quality of life among β-TM patients, emphasizing the necessity of comprehensive management strategies to safeguard patient well-being.

## Acknowledgments

Special thanks to Zhibin Xu, PhD, of the Guangzhou Institute of Respiratory Health, affiliated with the First Affiliated Hospital of Guangzhou Medical University, for his expert revision and language editing of our manuscript.

## Author contributions

**Software:** Xiang Lan, Lili Liu.

**Validation:** Xiang Lan, Linming Huang.

**Project administration:** Zhonglv Ye.

**Writing—original draft:** Zhonglv Ye.

**Writing—review & editing:** Zhonglv Ye.

**Formal analysis:** Jiayi Du.

**Data curation:** Chuan Tian.

**Resources:** Xiaohuan Mo.

**Visualization:** Xiaohuan Mo.
